# Cervical Artery Dissection in Autosomal Dominant Polycystic Kidney Disease

**DOI:** 10.3390/medicina62010019

**Published:** 2025-12-22

**Authors:** Anna Liu, Helena Xeros, Waseem Wahood, Zafer Keser, Muhib Khan

**Affiliations:** 1 Department of Neurology, Mayo Clinic Rochester, 200 First St. SW, Rochester, MN 55905, USA; 2 Department of Radiology, University of Miami/Jackson health System, 1400 NW 12th Ave, Miami, FL 33136, USA

**Keywords:** cervical artery dissection, autosomal dominant polycystic kidney disease, risk factors, national inpatient sample

## Abstract

*Background and Objectives*: Autosomal dominant polycystic kidney disease (ADPKD) is characterized by multisystem involvement, including renal cysts, hepatic cysts, intracranial aneurysms, and aortic root dilatation and dissection. Though exceedingly rare, cervical artery dissections (CeAD) have been reported in association with ADPKD. The aim of this retrospective observational study is to investigate clinical features in patients with ADPKD that increase the probability of an associated CeAD diagnosis. *Materials and Methods*: The National Inpatient Sample from 2016 to 2020 was utilized to compare clinical features for patients with an ICD-10 code diagnosis of ADPKD, CeAD, and both ADPKD and CeAD. The Cochran–Armitage test and Chi-square test were utilized to assess clinical features or trends in ADPKD patients associated with a concurrent CeAD diagnosis. *Results*: Between 2016 and 2020, there were 224,065 people with ADPKD, 86,135 with CeAD and 155 with both (0.05%). The total cohort had a mean age of 56.74 years, with 47.26% female participants (*p* = 0.70), and was predominantly white (66.15%, *p* < 0.001). In patients with ADPKD, comorbid acute ischemic stroke (*p* < 0.001), transient ischemic attack (*p* < 0.001), aortic dissection (*p* < 0.001), coronary artery dissection (*p* < 0.001), subarachnoid hemorrhage (*p* < 0.001), coagulation defects (*p* = 0.002), and hypertension (*p* < 0.001) are risk factors associated with an increased probability of concomitant CeAD. *Conclusions*: CeAD in ADPKD patients is rare. In ADPKD patients, acute ischemic stroke, transient ischemic attack, aortic dissection, coronary artery dissection, subarachnoid hemorrhage, coagulation defects, and hypertension are risk factors of concomitant CeAD. Recognizing these factors can aid in the decision to screen for concomitant CeAD in patients with ADPKD.

## 1. Introduction

Autosomal dominant polycystic kidney disease (ADPKD) is a multisystem disorder characterized by bilateral renal cysts, hypertension, renal insufficiency, and vascular abnormalities including aortic root dilatation, dissections, and intracranial aneurysms. It is the most common hereditary renal disease and the most common potentially lethal single-gene disorder [1]. Mortality ranges between 18.4 and 37.4 deaths per 1000 patient-years among ADPKD patients with chronic kidney disease (CKD) and end-stage renal disease (ESRD), respectively. ADPKD results from heterozygous pathogenic variants in PKD1 or PKD2, encoding polycystin-1 and -2, which regulate cystogenesis and vascular integrity [1,2]. Loss of polycystin function promotes cystic and vascular manifestations [1]. Age-specific imaging with supportive family history or genetic confirmation typically establishes diagnosis between ages 27 and 42, with prognosis influenced by genotype and disease complications [1,3,4,5].

Cervical artery dissection (CeAD) involves the internal carotid or vertebral arteries. It typically arises from an intimal tear or primary intramural hematoma. Subintimal extension of dissection causes stenosis or occlusion, whereas sub-adventitial involvement results in dissecting aneurysm formation. CeAD may be spontaneous, traumatic, or iatrogenic and although uncommon overall, represents a major cause of ischemic stroke in young adults [2,6].

Intracranial aneurysms are a well-documented vascular manifestation of ADPKD, while less common associations include thoracic aortic dissections, aortic root dilatation, dolichoectasia, and coronary artery dissections [1,3]. The co-occurrence of ADPKD and CeAD has been cited in the literature but is rare and mostly reported as a case report or series. The pathophysiology underlying the co-occurrence of ADPKD and CeAD has not been elicited. However, it should be noted that familial clustering has been seen with intracranial aneurysms and CeAD, raising the possibility of an inherited predisposition. It is thought that there may be a genetic propensity for arterial weakness leading to the development of vascular abnormalities [7].

Given this is an uncommon manifestation, there is limited information regarding clinical features in patients with ADPKD that increases the likelihood of a concurrent CeAD diagnosis and vice versa. The purpose of this retrospective observational study is to expand upon previous case-based literature by utilizing the National Inpatient Sample to determine the prevalence of comorbid ADPKD and CeAD and to define associated clinical features. Identifying demographic or clinical predictors may inform diagnosis, screening, and management in patients with either or both conditions.

## 2. Materials and Methods

We conducted a retrospective chart review using the National Inpatient Sample (NIS, https://hcup-us.ahrq.gov/news/exhibit_booth/nis_brochure.jsp, accessed on 19 May 2025) from 2016 to 2020 and analyzed various clinical features associated with patients diagnosed with ADPKD alone, CeAD alone, and both ADPKD and CeAD. The NIS database is maintained under the Healthcare Cost and Utilization Project (HCUP) and is sponsored by the Agency for Healthcare Research and Quality. It encompasses data from approximately 7 million hospital stays from over 1000 hospitals in the United States [8]. Data is obtained from the 48 states participating in HCUP and encompasses U.S. community hospitals, including public institutions and academic medication centers. Excluded centers include rehabilitation of long-term acute care hospitals. The NIS does not include patient identifiers and is therefore exempt from institutional review board approval [9,10].

Patient cohorts were identified from the National Inpatient Sample (NIS) database between 2016 and 2020, comprising individuals diagnosed with ADPKD alone, CeAD alone, or both conditions. NIS sampling weights were applied. Clinical data were extracted using International Classification of Diseases, Tenth Revision (ICD-10) codes corresponding to diagnoses and comorbidities associated with ADPKD (ICD-10 codes Q61.2, Q61.3, Q61.8, and Q61.9) and CeAD (ICD-10 codes I77.71, I77.74, and I77.75). These codes were listed as hospital diagnoses but were not necessarily the primary diagnosis. Investigated features were chosen based on clinical relevance and prior case-based literature. If a patient did not have the diagnosis code during their hospitalization, it was assumed that they did not have the condition since codes are validated. Associated conditions included acute ischemic stroke, transient ischemic attack, vertebrobasilar or carotid artery syndrome, vertebrobasilar dolichoectasia, arteriovenous malformation, intracranial aneurysm, carotid and vertebral stenosis, carotid and vertebral aneurysm, subarachnoid hemorrhage, aortic dissection, dilated aortic root, aortic ectasia, coronary artery disease and dissection, mitral valve prolapse, arterial dissection or rupture, atrial fibrillation, congestive heart failure, peripheral vascular disease, hypertension, hyperlipidemia, type 2 diabetes, hypothyroidism, nicotine dependence, long-term anticoagulant or antiplatelet/antithrombotic use, end-stage renal disease, renal insufficiency or failure, hepatic cyst, seminal cyst, liver disease, abdominal hernia, thrombophilia, coagulation defects, antiphospholipid syndrome, lupus anticoagulant, systemic lupus erythematous, fibromuscular dysplasia, connective tissue disease, hypermobility, immunodeficiency, coronavirus disease 2019, HIV/AIDs, lymphoma and metastatic cancer. In addition to these clinical features and comorbidities, imaging and interventions such as computed tomography angiography, magnetic resonance angiogram, fluoroscopy/digital subtraction angiography, coiling, clipping, and stenting were extracted from the NIS database [11].

The prevalence of the corresponding associated clinical features and comorbidities’ ICD-10 codes in patients with ADPKD alone, CeAD alone, and both ADPKD and CeAD were extracted for comparison and analysis. Categorical variables were documented as numbers and percentages while continuous variables were documented as means. To evaluate for statistical differences in the prevalence of associated conditions, Chi-square tests were used to compare categorical variables among patients with ADPKD, CeAD, and those with both conditions. Statistical differences in continuous variables were assessed using Student’s *t*-test, comparing two variables at a time (ex. ADPKD versus CeAD and ADPKD versus both CeAD and ADPKD). Trends across groups such as race and gender were evaluated using the Cochran–Armitage test. Statistical significance was defined as *p* < 0.05.

## 3. Results

### 3.1. Demographics

Between 2016 and 2020, there were a total of 224,065 people with a diagnosis of ADPKD, 86,135 people with CeAD, and 155 people with both. The mean age at the time of admission was 57.72 years for those with ADPKD, 54.21 years for those with CeAD, and 51.39 years for those with both. The mean age for all patients was 56.74 years. Within the total cohort, there was no statistical difference between males and females with 47.26% female (*p* = 0.70). The total cohort was predominantly white (66.15%, *p* < 0.001). Most patients were evaluated within urban teaching hospitals (80.02%) with Medicare being the most common payer (51.22%). Demographic data is included in Table 1.

### 3.2. Clinical Features and Comorbidities

**Figure 1:**
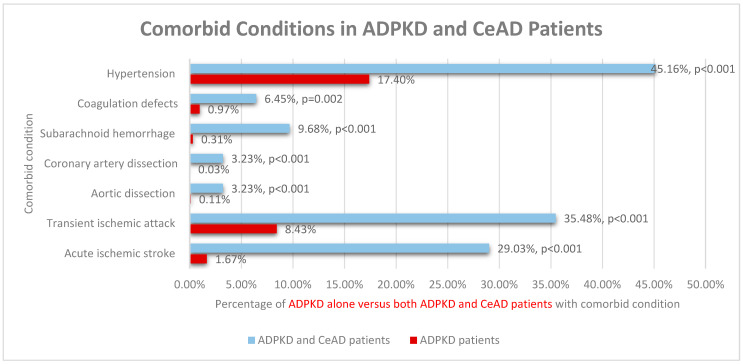
Comorbid Conditions in ADPKD and CeAD Patients. Prevalence of comorbid conditions in patients with ADPKD alone versus both ADPKD and CeAD.

In patients with ADPKD, comorbid acute ischemic stroke, transient ischemic attack, aortic dissection, coronary artery dissection, subarachnoid hemorrhage, or hypertension were risk factors associated with an increased probability of a concurrent CeAD diagnosis with *p* < 0.001 for all. Coagulation defects were also an associated risk factor with *p* = 0.002 (Figure 1).

**Figure 2:**
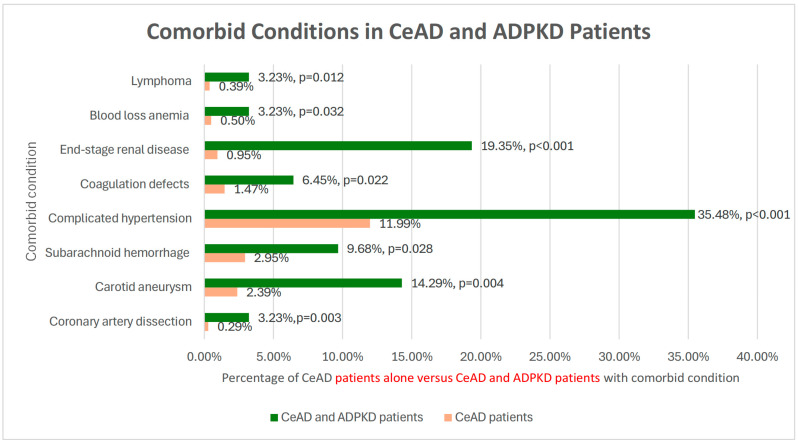
Comorbid Conditions in CeAD and ADPKD Patients. Prevalence of comorbid conditions in patients with CeAD alone versus both CeAD and ADPKD.

Conversely, in patients with CeAD, a concomitant diagnosis of coronary artery dissection (*p* = 0.003), carotid aneurysm (*p* = 0.004), subarachnoid hemorrhage (*p* = 0.028), complicated hypertension (*p* < 0.001), coagulation defects (*p* = 0.022), ESRD (*p* < 0.001), blood loss anemia (*p* = 0.032), or lymphoma (*p* = 0.012) carried an increased probability of associated ADPKD (Figure 2). There is a trend of concurrent CeAD and aortic dissection co-occurring with an associated ADPKD diagnosis, but this was not statistically significant. A list of all evaluated characteristics with corresponding *p*-values and ICD-10 codes can be found in Table 2. The table highlights elevated prevalences of vascular comorbidities such as acute ischemic stroke, transient ischemic attack, and hypertension/complicated hypertension in the ADPKD and CeAD group even when compared to other statistically significant features.

### 3.3. Treatment

Regarding interventions, CeAD patients requiring endovascular clipping for management of intracranial aneurysms had a higher likelihood of associated ADPKD (*p* < 0.001). Patients with ADPKD who underwent coiling or fluoroscopy/digital subtraction angiography were more likely to have concurrent CeAD (*p* < 0.001). With respect to current pharmacologic therapies, no significant association was observed between long-term use of antiplatelet or anticoagulant medications and a concurrent diagnosis of ADPKD or CeAD.

## 4. Discussion

In this retrospective observational study, we examined various associated clinical features of ADPKD and CeAD to evaluate comorbidities that are associated with a concurrent ADPKD or CeAD diagnosis. To our knowledge, the previous literature has been primarily case-based, making our study of associated clinical features unique given our large, national cohort. Data was derived from the National Inpatient Sample capturing a nationally representative sample of hospitalizations in the United States and showed that the presence of certain vascular, hematologic, renal, and oncologic comorbidities such as coronary artery dissection and hypertension can be associated with a comorbid ADPKD or CeAD diagnosis.

The prevalence of ADPKD at birth is approximately 1:1000 and approximately 300,000 people are affected in the United States [1]. It is therefore important to recognize clinical manifestations of the disease, many of which are vascular. More commonly recognized vascular manifestations include intracranial and cardiac aneurysms in addition to aortic dilatation and dissection [12,13]. Intracranial aneurysms are noted in approximately 9–14% of patients with ADPKD, with younger age (mean 39 years) compared to the general population. Previous studies have reported a five to nine-fold increased risk of aortic dissection compared to general population with higher incidence in patients with hypertension and progression of CKD [14]. However, it should be noted that dissections and aneurysms of almost every large artery have been documented [14]. Existing literature has noted the rare co-occurrence of ADPKD and CeAD but our study aims to build upon previous studies and define associated clinical features between the two conditions. To our knowledge, it is the largest study to date examining these associations with a goal of aiding clinical decision-making.

The wide array of vascular anomalies seen in ADPKD supports the hypothesis that polycystins are required to maintain vascular integrity. On a cellular level, both polycystin 1 and polycystin 2 are expressed in endothelial cells and vascular smooth muscle cells. However, there are differences in polycystin expression in organ systems with high levels of PKD1 being found in the cardiovascular system. In mice, homozygous mutations of PKD 1 or PKD 2 have resulted in embryonic mortality while mice that express a reduced amount of polycystin 1 survive but develop cysts. However, the mechanism in which PKD mutations increase the risk of vascular abnormalities is unknown. One possible explanation is that the development of vascular complications is dependent on focal and somatic loss of the wild-type PKD allele in vasculature. This is because endothelial cells heterozygous for PKD 2 mutations have demonstrated a normal response to shear stress, supporting that a two-hit mechanism is needed for the development of vascular anomalies. Clinically, patients with ADPKD-associated intracranial aneurysms have a rupture rate five times higher than the general population. In addition, the average age of rupture is ten years younger in the ADPKD population. As intracranial aneurysm rupture results in significant morbidity and mortality, knowledge of these associations is paramount to facilitate early detection and intervention [14]. By defining these associations, our study shows that the presence of certain clinical features in ADPKD patients such as acute ischemic stroke, aortic and coronary artery dissections, and coagulation defects can be associated with CeAD and thus, screening should be considered.

Few cases of CeAD associated with ADPKD have been reported as compared to other vascular abnormalities, highlighting the rarity of occurrence [15,16]. Review of the literature shows that when they do occur, presenting symptoms include headache, cervicalgia, hemiparesis, vomiting, and dizziness. Many patients have a history of hypertension, and a fewer number of patients have pre-existing CKD. Evaluation at times also revealed the presence of intracranial aneurysms but this was not universal [4,5,6,7,13,15]. Bobrie et al. noted that when arterial dissections do occur, patients have documented dissections in multiple vessels, recurrent dissections, concurrent aneurysms, or family members with vascular complications [7]. In non-ADPKD patients, five percent of those with CeAD have a positive family history. Schievink et al. noted three families with both intracranial aneurysms and CeAD. This brings up the likelihood of a predisposition for diffuse vascular disease if one condition is already present or if there is a family history [12]. It is possible that the development of a dissection is the result of genetic predisposition to vascular complications followed by a somatic second hit. However, the exact pathophysiology is poorly understood and requires additional investigation.

Hypertension, progression of CKD, presence of intracranial aneurysm, male sex, older age, and family history of vascular disease have been identified as risk factors for arterial dissection in ADPKD. However, most of the literature pertains to aortic dissection [17,18,19]. In our study, aortic dissection, coronary artery dissection, and hypertension were found to be risk factors of concomitant CeAD, suggesting potentially similar underlying pathophysiology of vascular compromise. Review of treatment modalities for CeAD in ADPKD patients showed that full dose aspirin was implemented in cases where patients had intracranial dissections or aneurysms while anticoagulation was favored in the absence of these features [7,15].

There is no current consensus on screening for potential CeAD in patients with ADPKD. The Kidney Disease: Improving Global Outcomes (KDIGO) 2025 clinical practice guideline was recently published with recommendations for intracranial aneurysm screening. Recommendations included screening for patients with ADPKD and a personal history of subarachnoid hemorrhage, family history of intracranial aneurysms, subarachnoid hemorrhage, or sudden explained death. Discussions regarding screening were also recommended for those with de novo ADPKD, unclear family history, and in those with a personal or family history of vascular disease. Time-of-flight magnetic resonance angiography or computed tomography angiography are the recommended methods of screening [20,21]. Although intracranial aneurysms are more prevalent than CeAD, the associated clinical features identified in our study may help inform and refine existing screening recommendations. While our findings do not elucidate why CeAD occurs less frequently in patients with ADPKD compared to other vascular abnormalities, they do underscore the importance of comorbid conditions that may predispose a patient to CeAD. Recognition of these comorbidities should inform clinical decision-making regarding vascular screening in this population.

Our study has limitations. Reliance on ICD-10 codes from inpatient records introduces potential misclassification (due to possible incorrect coding), selection bias (as outpatient cases were not captured), and surveillance bias (given that patients with ADPKD may undergo more frequent imaging by which CeAD may be identified). The analysis identified associations between ADPKD and CeAD but cannot quantify risk, define underlying mechanisms, or determine incidence or absolute risk. Detailed information such as radiographic features, timing of diagnoses, severity of disease, and genetics were not investigated. The study rather identifies patterns of co-occurring clinical features in hospitalized patients. Future studies integrating both inpatient and outpatient populations and incorporation imaging data are needed to better characterize the pathophysiologic and radiographic features underlying their co-occurrence.

## 5. Conclusions

Our retrospective observational study identified associated clinical features for CeAD in ADPKD patients utilizing the National Inpatient Sample, which accounts for hospitalized patients. It highlights that vascular comorbidities such as hypertension/complicated hypertension, subarachnoid hemorrhage, and coronary artery dissection in addition to hematologic, renal, and oncologic comorbidities are can be associated with concurrent diagnoses of ADPKD or CeAD. This information can guide screening for concurrent CeAD in patients with ADPKD and vice versa.

## Figures and Tables

**Table 1:** Patient Demographics.

	Autosomal Dominant Polycystic Kidney Disease (*n* = 224,065)	Cervical Artery Dissection (*n* = 86,135)	Autosomal Dominant Polycystic Kidney Disease and Cervical Artery Dissection (*n* = 155)	Total (*n* = 310,355)	*p*-Value
Age, mean	57.72 years	54.21 years	51.39 years	56.74 years	
Female	48.64%	43.68%	51.61%	47.26%	0.70
**Race**					
White	64.48%	70.54%	82.14%	66.15%	<0.001
Black	18.41%	12.77%	7.14%	16.86%	
Hispanic	11.09%	9.62%	3.57%	10.68%	
Asian/Pacific Islander	2.72%	3.47%	0.00%	2.92%	
Native American	0.54%	0.52%	7.14%	0.54%	
Other	2.77%	3.07%	0.00%	2.85%	
**Primary expected payer**					
Medicare	58.98%	31.02%	38.71%	51.22%	
Medicaid	13.13%	16.30%	9.68%	14.01%	
Private insurance	23.25%	42.28%	45.16%	28.53%	
Self-pay	2.32%	5.99%	6.45%	3.34%	
No charge	0.21%	0.39%	0.00%	0.26%	
Other	2.11%	4.01%	0.00%	2.64%	
**Location/teaching status of hospital**					
Rural	5.42%	2.04%	0.00%	4.48%	
Urban, nonteaching	17.60%	10.03%	9.68%	15.50%	
Urban, teaching	76.98%	87.93%	90.32%	80.02%	

Demographic data of evaluated cohort including patients with ADPKD alone, CeAD alone, both ADPKD and CeAD, and the total patient cohort.

**Table 2:** Patient Characteristics and ICD-10 Codes.

	Autosomal Dominant Polycystic Kidney Disease (ADPKD)	Cervical Artery Dissection (CeAD)	ADPKD and CeAD	*p*-Value for ADPKD Versus both ADPKD and CeAD	*p*-Value for CeAD Versus both ADPKD and CeAD	ICD-10 Code
**Statistically** **Significant** **Characteristics**						
Acute ischemic stroke	1.67%	44.91%	29.03%	<0.001	0.076	I63, I67.82, I67.89, I69.79
Transient ischemic attack	8.43%	50.36%	35.48%	<0.001	0.099	G45.9
Aortic dissection	0.11%	0.63%	3.23%	<0.001	0.068	I71.00
Coronary artery dissection	0.03%	0.29%	3.23%	<0.001	0.003	I25.42
Subarachnoid hemorrhage	0.31%	2.95%	9.68%	<0.001	0.028	I60.9
Hypertension	17.40%	45.89%	45.16%	<0.001	0.935	I10
Complicated hypertension	64.88%	11.99%	35.48%	<0.001	<0.001	I1A.0
Coagulation defects	0.97%	1.47%	6.45%	0.002	0.022	D68.9
Carotid aneurysm	0.00%	2.39%	14.29%	N/A	0.004	I72.0
(ESRD)	41.08%	0.95%	19.35%	0.014	<0.001	N18.6
Blood loss anemia	1.28%	0.50%	3.23%	0.336	0.032	D50.0
Lymphoma	1.08%	0.39%	3.23%	0.247	0.012	C85.80
Clipping	0.07%	0.12%	3.23%	<0.001	<0.001	03VG0CZ. 03VG0ZZ
Coiling	0.26%	3.18%	6.45%	<0.001	0.300	03VG3[B,DJZ
Fluoroscopy/digital subtraction angiography	0.45%	14.90%	12.90%	<0.001	0.756	B31[3,4,5,6,7,8,B, C,D,F,G,H,R] [0,1,Y]Z
**Not statistically** **significant patient characteristics**						
Vertebrobasilar syndrome	0.01%	0.27%	0.00%	0.953	0.775	G45.0
Carotid artery syndrome	0.00%	0.10%	0.00%	N/A	0.865	G45.1
Vertebrobasilar dolichoectasia	0.01%	0.20%	0.00%	0.964	0.802	Q28.3
Arteriovenous malformation	0.03%	0.19%	0.00%	0.923	0.806	Q28.2
Intracranial aneurysm	1.07%	4.15%	3.23%	0.245	0.796	I67.1
Vertebral stenosis	0.00%	12.14%	5.88%	N/A	0.430	I65.0
Carotid stenosis	0.00%	21.24%	28.57%	N/A	0.503	I65.2
Vertebral aneurysm	0.00%	3.03%	5.88%	N/A	0.495	I72.6
Dilated aortic root	0.09%	0.10%	0.00%	0.873	0.910	I72.0
Aortic ectasia	0.08%	0.10%	0.00%	0.873	0.910	I77.819
Coronary artery disease	22.02%	12.13%	3.23%	0.012	0.129	I25.1
Mitral valve prolapse	0.49%	0.34%	0.00%	0.697	0.746	I34.1
Arterial dissection	0.31%	1.82%	0.00%	0.756	0.453	I77.79
Arterial rupture	0.01%	0.11%	0.00%	0.953	0.855	I77.2
Atrial fibrillation	8.43%	4.50%	3.23%	0.297	0.733	I48.0, I48.1
Congestive heart failure	25.25%	8.93%	3.23%	0.005	0.266	I50.20
Peripheral vascular disease	10.47%	9.88%	19.35%	0.107	0.078	I73.9
Hyperlipidemia	5.24%	4.68%	6.45%	0.761	0.640	E78.0
Type 2 diabetes	4.65%	8.07%	6.45%	0.633	0.741	E11.9
Hypothyroidism	11.81%	7.85%	6.45%	0.355	0.773	E03.9
Nicotine dependence	2.12%	3.73%	0.00%	0.410	0.272	F17.200, Z87.891
Long-term anticoagulant use	10.07%	7.41%	6.45%	0.503	0.838	Z79.01
Long-term antiplatelet/antithrombotic use	16.29%	21.26%	16.13%	0.981	0.492	Z79.02, Z79.82
Renal insufficiency or failure	0.80%	0.42%	0.00%	0.614	0.720	N28.9
Hepatic cyst	3.62%	0.15%	0.00%	0.276	0.833	K76.89
Seminal cyst	0.17%	0.05%	0.00%	0.815	0.898	N50.89
Liver disease	10.08%	2.82%	0.00%	0.0600	0.341	K72.90
Abdominal hernia	0.53%	0.10%	0.00%	0.685	0.863	K43.9
Thrombophilia	0.17%	0.17%	0.00%	0.830	0.817	D68.69
Antiphospholipid syndrome	0.15%	0.17%	0.00%	0.831	0.817	D68.61
Lupus anticoagulant	0.12%	0.09%	0.00%	0.851	0.867	D68.62
Systemic lupus erythematous	0.54%	0.42%	0.00%	0.680	0.714	M32.9
Fibromuscular dysplasia	0.00%	0.85%	0.00%	0.979	0.604	I77.3
Connective tissue disease	0.02%	0.09%	0.00%	0.944	0.868	M35.89
Hypermobility	0.01%	0.00%	0.00%	0.964	N/A	M35.7
Immunodeficiency	0.27%	0.04%	0.00%	0.772	0.910	D84.9
Coronavirus disease 2019	0.02%	0.00%	0.00%	0.937	N/A	B34.2
HIV/AIDS	0.30%	0.21%	0.00%	0.761	0.796	B20
Metastatic cancer	2.18%	0.85%	0.00%	0.401	0.607	C79

A list of all investigated clinical features in this study with corresponding ICD-10 codes and *p*-values. N/A corresponds to not applicable.

## Data Availability

The data presented in this study are available the National Inpatient Sample (NIS, https://hcup-us.ahrq.gov/news/exhibit_booth/nis_brochure.jsp, accessed on 16 December 2025). These data were derived from the following resources available in the public domain: the National Inpatient Sample (NIS, https://hcup-us.ahrq.gov/news/exhibit_booth/nis_brochure.jsp, accessed on 16 December 2025). The datasets analyzed for this study are included in the article. Please reach out to the corresponding author regarding additional information.

## Abbreviations

The following abbreviations are used in this manuscript:

ADPKD	Autosomal dominant polycystic kidney disease
CeAD	Cervical artery dissection
CKD	Chronic kidney disease
ESRD	End-stage renal disease
HIV/AIDS	Human immunodeficiency virus/acquired immunodeficiency syndrome
